# Pulmonary Involvement in Acute Rheumatic Fever: A Case Report and Literature Review

**DOI:** 10.7759/cureus.7295

**Published:** 2020-03-16

**Authors:** Patrick Kenney, Sarah McMullin, Rabheh Abdul-Aziz

**Affiliations:** 1 Department of Medicine, Jacobs School of Medicine and Biomedical Sciences, University at Buffalo, Buffalo, USA; 2 Department of Pediatrics, Jacobs School of Medicine and Biomedical Sciences, University at Buffalo, Buffalo, USA; 3 Department of Pediatric Rheumatology, Jacobs School of Medicine and Biomedical Sciences, University at Buffalo, Buffalo, USA

**Keywords:** acute rheumatic fever, rheumatic heart disease, carditis, rheumatic pneumonia, rheumatic fever, rheumatic pneumonitis

## Abstract

Rheumatic pneumonia is a pulmonary complication of rheumatic fever, often with grave outcomes. It has been described sporadically in literature, most recently a decade ago. Here, we describe a case of a 12-year-old Native American girl presenting with chest pain, gastrointestinal complaints, and frequent nosebleeds. After the initial diagnosis with acute pericarditis, she was found to meet diagnostic criteria for rheumatic fever. Revised Jones criteria met included significantly elevated streptolysin O antibody and anti-DNase B, carditis, arthralgia, fever, and elevated inflammatory markers. Findings complicating the diagnosis were an elevated antinuclear antigen with a family history of systemic lupus erythematosus (SLE), hemoptysis, and a chest CT finding of right lower lobe alveolar hemorrhage as well as right-sided mediastinal adenopathy. The patient was discharged on day nine of admission after a course of high-dose methylprednisolone with prednisone taper, furosemide, enalapril, naproxen, monthly penicillin G injections, and multidisciplinary outpatient follow-up. A repeat chest CT scan three months later showed significant improvement. The pulmonary findings described in our patient are consistent with prior reports of rheumatic pneumonia, however, most prior cases described did not include high-resolution imaging. Our patient recovered well aside from complications secondary to mitral regurgitation, unlike many patients seen in our literature search who died due to early or later complications of pulmonary disease. Although acute rheumatic fever, and its pulmonary complications, is significantly less common than it once was, it remains a disease entity that should remain on the differential for multisystem rheumatic complaints.

## Introduction

Rheumatic fever was first described in the late 1600s by Sydenham as a differentiation between gout and rheumatoid arthritis [1]. For over 200 years, not much changed in our understanding of the disease until bacteriologic advances in the late 1800s helped identify a correlation between streptococcal pharyngitis and eventual onset of rheumatic fever [1]. The discovery of anti-streptolysin O in the 1930s revolutionized the diagnosis and helped fully differentiate acute rheumatic fever (ARF) from other arthritides [1]. Following the development of the sulfonamides and penicillins in the late 1930s and their use in the prevention of ARF in the 1940s, antibiotics quickly became the mainstay of therapy [2]. There remains robust discussion on optimal doses, durations, and types of supplemental therapies, but long-term penicillin remains the most common therapy prescribed to prevent the recurrence of rheumatic fever [1-2].

The prevalence of acute rheumatic fever has declined significantly since the introduction of modern antibiotics [3]. Current estimates range from 0.6 to 3.7 cases per 100,000 per year in the United States, with higher rates in Hawaii and American Samoa [4-5]. That said, it is a diagnosis that should remain on the differential for clinicians in the outpatient, emergency, and inpatient settings primarily due to the morbidity associated with untreated group A streptococcus (GAS) infections. The modern diagnostic criteria were most recently updated in 2015 with the Revised Jones criteria [6]. In the text below, we describe an atypical presentation of advanced rheumatic fever with pulmonary complications consistent with prior reports of rheumatic pneumonia.

## Case presentation

A 12-year-old premenstrual female of Native American ancestry with mild intermittent asthma and recurrent self-resolving nosebleeds for the past several years presented to an outside institution with sharp, left-sided constant chest pain worsening on deep inspiration accompanied by shortness of breath starting the day of presentation. She also reported a five to six-day history of nausea, vomiting, diarrhea, and fevers as high as 103°F the day prior, two months of worsening polyarthralgias, weight loss of 5 kilograms (10% body weight), and malaise. There was a family history of SLE in her paternal aunt and her mother was being followed by rheumatology for unspecified arthritis.

She was initially diagnosed with pericarditis based on classic electrocardiogram (EKG) findings, including sinus tachycardia, ST elevations, and PR depressions throughout, with a significantly elevated erythrocyte sedimentation rate (ESR). She was transferred to our tertiary children's hospital for further management. Vital signs on arrival were significant for persistent tachycardia and intermittent hypoxemia requiring oxygen supplementation. She remained afebrile and with stable blood pressures. Physical exam during the first 24 hours of her stay demonstrated a friction rub and signs of fluid overload with pretibial pitting edema and jugular venous distention. Initial workup showed an elevated erythrocyte sedimentation rate (ESR) of 136 mm/hr (normal <20 mm/hr), and significant microcytic anemia with hemoglobin of 7.3g/dL (last checked at the age of four, when it was normal). Significant laboratory findings are noted in Table 1.

**Table 1:** Pertinent positive and negative laboratory findings from early in the hospital course Days refer to the hospital day on which samples were drawn; results were available as described in the body of the article. Ab - Antibody, ENA - Extractable Nuclear Antigen, C-ANCA - Cytoplasmic antineutrophil cytoplasmic antibodies, P-ANCA - Perinuclear antineutrophil cytoplasmic antibodies

	Normal range			Presentation	Day 1	Day 2
Leukocytes	4.0	to 10.5	10^9/L	16.2		
Hemoglobin	12.0	to 15	g/dL	7.3		
Platelets	150	to 450	10^9/L	440		
ESR	0	to 20	mm/hr	136		
CRP	0.2	to 10	mg/L	157		
ANA - Nuclear Ab Titer		<1:40			1:1280	
DNA Double Strand Ab		Negative			Negative	
Streptolysin O Ab	0	to 199	unit/mL			2490
Anti Dnase B Titer	0	to 376	unit/mL			626
Cardiolipin Abs Qualitative						Negative
Cardiolipin Ag IgG						Within normal limits
Cardiolipin Ag IgM						Within normal limits
Dilute Russell Viper Venom Ratio	0	to 1.19	units			1.22
Beta 2 Glycoprotein 1 Ab						Negative
Rheumatoid Factor						Within normal limits
RNP Extractable Nuclear Ab						Negative
Smith Extractable Nuclear Ab						Negative
Sjogrens Syndrome-A ENA						Negative
Sjogrens Syndrome-B ENA						Negative
Histone Ab IgG						Negative
C3						Within normal limits
C4						Within normal limits
IgA	70	to 390	mg/dL			622
IgG	680	to 1531	mg/dL			2450
IgM	50	to 300	mg/dL			145
Myeloperoxidase Ab IgG						Within normal limits
C-ANCA						Negative (<1:10)
P-ANCA						Negative (<1:10)
Atypical P-ANCA						Negative (<1:10)
Serine Proteinase Ab IgG						Within normal limits
Angiotensin Converting Enzyme						Within normal limits
Blastomyces Ab						Negative

A subsequent transthoracic echocardiogram demonstrated increased echogenicity of the pericardium without evidence of pericardial effusion, consistent with pericarditis. Findings were also significant for moderate mitral regurgitation with associated significant left atrial enlargement, tricuspid regurgitation, trivial aortic insufficiency, and moderate pulmonary hypertension with right ventricular systolic pressures 36 cm above that of the right atrium. There were no signs of vegetations, and her ejection fraction was preserved. Renal ultrasound with Doppler performed due to proteinuria of 22 mg/dL (normal 1-14 mg/dL) showed no signs of intrinsic disease.

She was transfused one unit of packed red blood cells for her anemia. Musculoskeletal and chest pain was managed initially with ketorolac and later with naproxen for their concurrent anti-inflammatory effects. Furosemide and enalapril were started for preload and after-load reduction with gradual resolution of fluid overload over 24 to 48 hours. During this period, she had several episodes of small-volume hemoptysis that improved in conjunction with diuresis. Given the clinical and laboratory findings as well as mitral valve involvement without a prior history of cardiac disease, there was a concern for an underlying infectious or inflammatory process.

**Figure 1:**
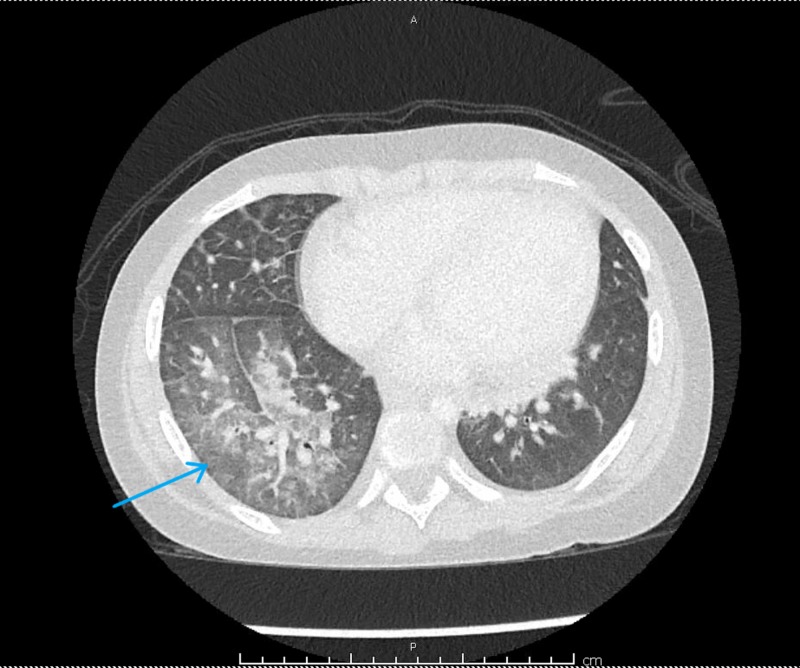
CTA chest Diffuse inflammatory changes with alveolar hemorrhage, as well as ground-glass opacities, noted in the right middle and lower lobes with left lower lobe atelectasis and hilar lymphadenopathy. CTA - Computed Tomography Angiogram

Rheumatology was consulted on day two of admission. There was a concern for possible vasculitis versus an atypical presentation of rheumatic fever. Workup was extended to include chest CT that showed cardiac findings consistent with the echocardiogram but also showed right-sided lung abnormalities including, centrilobular ground-glass, intralobular septal thickening, and alveolar opacification consistent with hemorrhage, as shown in Figure 1. These findings were less severe in the right upper lobe and worse in the right middle and lower lobes. There was associated right-sided hilar and mediastinal adenopathy. Pulmonology evaluated the patient for possible bronchoscopy, but as hemoptysis was already improving, this was deferred. While lab results were pending, given the significant multiorgan involvement, she was started on high-dose methylprednisolone daily for three days with conversion to oral prednisone taper thereafter. Azithromycin was started as prophylaxis against atypical infections while undergoing the initial high-dose steroid course. A more extensive infectious disease workup was not undertaken due to a lack of evidence for another source after initial labs and imaging. She had good clinical improvement, with a resolution of tachycardia and hypoxemia, as well as a significant improvement in her chest pain.

On days three and four of admission, the first rheumatologic lab results returned significant for significantly positive antinuclear antibodies (ANA) and elevated streptolysin O Ab and anti-DNase B. Other pertinent rheumatologic studies (all negative) are also shown in Table 1. Throat culture was performed, which was negative but was collected after starting azithromycin. Penicillin G was initiated for the treatment of rheumatic fever with the first dose on day five of admission.

The patient continued to improve and was discharged on day nine on admission with home nursing support and close outpatient multispecialty follow-up. She was discharged on furosemide, enalapril, naproxen, and monthly penicillin injections. Her post-discharge course included multiple emergency department visits for recurrent chest pain but this has gradually improved. Her family was tested and treated prophylactically with amoxicillin as her brother, sister, and mother were found to be group A strep carriers.

She has continued to follow with cardiology and rheumatology. Blood work after discharge showed the normalization of ESR within four weeks and a gradual reduction in her antistreptolysin O (ASO) titer. Repeat chest CT scan three months after presentation demonstrated a resolution of ground-glass and right-sided consolidations with minimal residual left lower lobe atelectasis and resolving lymphadenopathy. An echocardiogram performed five months after her initial presentation showed mild mitral regurgitation, mild aortic valve insufficiency, normalization of left heart chamber size, no pulmonary hypertension, and normal biventricular function. She has not had further episodes of fluid overload and has tolerated gradual reduction in her diuretic. She will continue to receive monthly penicillin G injections until the age of 18.

## Discussion

We offer this case of rheumatic pneumonia as an example of a rapidly progressive case of ARF with multiorgan involvement. The most recent report of this entity from a search of Ovid MedLine and Pubmed is from 2005 with seven English-language reports in the preceding two decades (Table 2) [7-16]. Our patient's presentation was severe and somewhat atypical, as there was significant anemia with a history of recent weight loss. She also reported non-migratory arthralgias of her shoulder, elbows, wrists, back, hips, and knees for two months prior to presentation. In addition, an elevated IgG at 2450 units/mL with a positive ANA supports an autoimmune process. These could suggest a previous episode or episodes of ARF that went without a diagnosis. In this case, her current presentation would be consistent with a recurrent acute episode exacerbating subacute or chronic changes. Throat culture was negative in our patient though it was obtained after antibiotics were administered. However, she did have elevated ASO and anti-DNase B titers and multiple culture-positive first-degree contacts in the same household. In low-risk populations, either ASO titers or throat culture data should guide diagnosis if there are other equally likely entities. In our patient, an elevated ANA initially, along with a history of oral ulcers, hemoptysis, and proteinuria, complicated diagnosis, but lupus and a vasculitis workup were otherwise negative and rapid improvement of diffuse symptomatic carditis and resolution of pulmonary findings were more consistent with ARF. Autopsy reports in earlier series have shown interstitial infiltrates, alveolar hemorrhage, and other inflammatory changes as were seen on CT chest in this report [16-17]. Close multispecialty observation over 18 months has not yielded a different diagnosis, and follow-up rheumatologic testing was negative. Prompt initiation of symptomatic treatment for cardiac dysfunction, the addition of pulse steroids, as well as the initiation of penicillin when ASO returned positive are believed to have been crucial in the improvement noted in this patient’s clinical course. Most large-scale studies are from the 1950-1960s, and do not show benefit from steroid administration but have significant inter-study variability [18-19]. The initiation of these therapies is thus empiric and guided by a patient's clinical course.

**Table 2:** Cases of rheumatic pneumonia found in a search of medical literature in the English language after 1975 References [7-16] ARF - Acute Rheumatic Fever, ASO (T.U.) - Antistreptolysin O, Todd Units

Year	Age	Sex	Location	ARF episode	Lung involvement	Steroid use	Steroid course	Throat culture	ASO (T.U.)	anti-Dnase B	Result
2005	13	Female	Qatar	First	Right	Yes	Not reported	Not reported	250	Not performed	Survived
2002	3	Female	Pernambuco, Brazil	First	Right	Prednisone	1 mg/kg daily	Not reported	600	Not performed	Survived
2001	18	Male	Spain	Second	Left lower lobe	Prednisone	60 mg daily, 2 month taper	No growth	400	Not performed	Survived
1995	19	Male	Utah, US	First	Right	Prednisone	1.5 mg/kg daily, taper not reported	No growth	400	1:340	Survived
1991	Less than 6	Not reported	Mexico	First - 2 cases; Second - 11 cases	13 cases, variable	Not reported	Not reported	Not reported	Not reported	Not reported	9 of 13 fatal
1990	10	Female	Ethiopia	Not reported	Right	Not reported	Not reported	Not reported	Not reported	Not reported	Death
1987	10	Male	Hawaii, US	First	Left lower lobe	No	None	Not reported	1600	Not performed	Survived
1985	14	Male	Israel	Second	Bilateral	Prednisone	60mg daily, tapered over >1 month	No growth	833	Not performed	Survived
1982	Not reported	Not reported	Romania	Not reported	6 cases, variable	Not reported	Not reported	Not reported	Not reported	Not reported	6 of 6 Survived
1975	13	Male	Arizona, US	First	Bilateral	Hydrocortisone	80 mg every 6 hours	Not reported	333	Not performed	Death

The limitations to this report include the absence of throat culture prior to the initiation of antibiotics as well as the absence of a lung biopsy.

## Conclusions

Rheumatic pneumonia is poorly understood. In recorded cases of rheumatic pneumonia, the pulmonary manifestations vary from pulmonary congestion out of proportion to observed left heart failure, infiltrative and consolidative processes, to alveolar hemorrhage and even the need for mechanical ventilation. Necropsy reports after fulminant cases have reported various degrees of necrosis and Masson bodies indicative of fibrotic changes. Although there can be a significant pulmonary disease, initiation of penicillin with or without high-dose steroids (as we utilized in the case above), addressing cardiac manifestations with diuretics and afterload reduction, and symptomatic care typically lead to significant improvement. Other than the use of penicillin as a secondary prophylaxis against future episodes, there remains significant debate on the benefit of other therapies. In our patient, her repeat CT chest only three months after presentation demonstrated the resolution of ground-glass opacities and resolving mediastinal adenopathy. Though she still has cardiac sequelae resulting from her carditis, her pulmonary findings (including pulmonary function testing) and symptoms have completely resolved. More prospective studies are needed to understand the presentation, severity, and outcome of pulmonary manifestations in patients with acute rheumatic fever.
